# Expression Pattern of the Cancer Stem Cell Marker “Nestin” in Leukoplakia and Oral Squamous Cell Carcinoma

**DOI:** 10.5041/RMMJ.10378

**Published:** 2019-10-29

**Authors:** Kushbu Narender Singh, Madhavan Nirmal Ramadas, Veeravarmal Veeran, Murali Radhakrishnan Naidu, Thamarai Selvi Dhanaraj, Kalyani Chandrasekaran

**Affiliations:** Division of Oral and Maxillofacial Pathology, Rajah Muthiah Dental College & Hospital, Annamalai University, Tamilnadu, India

**Keywords:** Biomarker, carcinoma, keratinocytes, leukoplakia, neoangiogenesis, nestin

## Abstract

**Objective:**

The aim of the present study was to determine and compare the expression pattern and localization of nestin, in an attempt to explore its role in oral carcinogenesis.

**Methods:**

Western blot and immunohistochemistry analysis were performed to study the expression pattern of nestin in normal mucosa, leukoplakia, and oral squamous cell carcinoma samples. Nestin expression was evaluated in the keratinocytes and blood vessels of all the samples and compared with various clinico-pathological parameters.

**Results:**

Nestin expression was increased in samples of leukoplakia and oral squamous cell carcinoma when compared with normal mucosa. Among leukoplakia samples, the expression was increased in cases without dysplasia compared to cases with dysplastic features. In cases of oral squamous cell carcinoma, the expression of nestin was found to be decreased with the loss of differentiation. Neoangiogenesis status determined by nestin expression showed an increasing expression from normal mucosa through leukoplakia, to oral squamous cell carcinoma.

**Conclusion:**

This study has two major findings: (1) identification of nestin as an effective indicator of neoangiogenesis, and (2) nestin may be used as a marker in predicting the early changes in oral carcinogenesis.

## INTRODUCTION

The incidence of oral cancer worldwide is around 500,000 new cases every year, accounting for approximately 3% of all malignancies, thus creating a significant health problem.^1^ The scenario is worsening with the 5-year survival rates dropping to 50%, persistent treatment failures, and frequent relapse/recurrence with exceptionally high morbidity and mortality rates.^2^ High oral cancer mortality rates are largely attributed to late-stage diagnosis.^3^

Oral potentially malignant disorders (PMD) are clinically significant because a majority of oral squamous cell carcinomas (OSCC) develop from these lesions.^4^ Oral leukoplakia is the most common PMD, and studies have shown that 17%–25% of oral leukoplakia lesions contain oral epithelial dysplasia, and about 8% progress to OSCC.^5^,^6^ Presently, there are no molecular markers available which enable us to distinguish lesions that may progress to OSCC from those that do not.

Recently, a great deal of attention has been directed at studying the cancer stem cell (CSC) population in tumors, which is thought to be the “roots of cancer.” Experimental evidence supports the cancer stem cells model in OSCC, which has spurred intense support in its clinical utility and has emerged as a new conceptual framework for OSCC treatment. The identification of this highly tumorigenic cell population could contribute to the development of novel therapeutic strategies and thus help mitigate the morbidity and mortality associated with cancer.^7^,^8^

Nestin is a class VI intermediate filament (IF) originally described as a neuronal stem cell marker during central nervous system development. It is also expressed in non-neuronal embryonic cells of skeletal muscle, heart, testis, pancreas, tooth bud, skin, hair follicle, and blood vessels.^9^ Cellular differentiation downregulates nestin expression which, in turn, is replaced by tissue-specific intermediate filaments. Brief expression of nestin at a very specific time and location suggests that nestin has a specific function in allowing cells to transition through these processes.^10^ Interestingly, nestin is re-expressed during pathological conditions such as in injury and regenerating tissues. Furthermore, nestin expression has also been reported in a wide array of tumors including breast cancer, lung cancer, glioma, melanoma, and pancreatic cancer. Increased expression of nestin is reported to correlate with aggressive growth, metastasis, and poor prognosis in many human tumors.^11^–^16^

Numerous studies have shown that nestin is known to contribute to the disassembly of vimentin during mitosis,^17^ inactivate cyclin-dependent kinase 5,^18^ and modulate mitosis-associated cytoplasmic reorganization during mitosis by CDC2 kinase.^19^

Nestin has also been implicated in angiogenesis, as evidenced by the observation of nestin expression in newly formed vessels but not in mature vasculature in various tumors. Accurate estimation of microvascular density (MVD) using nestin in tumors has revealed a strong relationship between tumor vessels and clinical outcome. These findings suggest that nestin is an effective marker for tumor angiogenesis and vascular formation in metastatic tumors.^20^

Many studies have evaluated the implication of nestin in human cancers and proved it to be a reliable biomarker. However, the exact role of this protein in OSCC is still uncertain. Hence, the present study was undertaken to evaluate the expression of nestin in the neoplastic progression of oral epithelium and to determine its role in the tumorigenesis of OSCC.

## MATERIALS AND METHODS

### Samples

Eight each of fresh OSCC and normal oral mucosa (NOM) samples were used for western blot analysis. An independent set of 10 NOM, 26 leukoplakia (leukoplakia samples without dysplasia, *n=*10; with mild/moderate dysplasia, *n*=10; with severe dysplasia, *n*=6), and 30 OSCC (well differentiated OSCC, *n*=10; moderately differentiated OSCC, *n*=10; poorly differentiated OSCC, *n*=10) samples were used for immunohistochemical analysis. All OSCC presented with primary tumor and had not undergone chemotherapy or radiotherapy before surgery. Ten NOM samples were gingival tissues harvested from patients undergoing prophylactic orthodontic surgery. The tissue samples and clinical data were obtained from the records of Rajah Muthiah Dental College & Hospital, Annamalai University. The study was approved by the Institutional Human Ethics Committee, Annamalai University, India (IHEC/0180/2016).

### Western Blot Analysis

The tissue samples were minced and, to 5 mg of tissue, 300 μL of lysis buffer was added and homogenized in radio immunoprecipitation assay buffer in an electric homogenizer. Then 1.5 mL of the homogenate was transferred to centrifuge tubes and centrifuged (12,000 rpm for 15 minutes at 4°C), reduced, and denatured. Extracts were subjected to 12% sodium dodecyl sulfate-polyacrylamide gel electrophoresis, which was run at 80 V for 1 hour. After electrophoresis, the proteins were electro-blotted to a polyvinylidene difluoride (PVDF) membrane by using 120 mA for 1 hour. Non-specific binding of antibody was blocked with 5% non-fat milk for 6 hours, and the sheet was incubated overnight at 4°C with mouse monoclonal anti-nestin antibody (Santa Cruz Biotechnology, Santa Cruz, CA, USA) at a dilution of 1:100. The membranes were washed with tris buffered saline with tween (TBST) thrice at 10 minutes’ interval and incubated with secondary antibody at a dilution of 1:2000. The PVDF membranes were washed again with TBST, and the developed bands were visualized by an enhanced chemiluminescence substrate.

### Immunohistochemistry

Sections of 4 μm thickness obtained from each paraffin block were taken on APES (3-aminopropyltriethoxysilane)-coated slides and used for nestin immunostaining. The sections were deparaffinized in xylene and rehydrated through graded alcohols, and the antigen retrieval was done by heating the slides in citrate buffer (pH 6.0) in a pressure cooker. The endogenous peroxidase activity was quenched by placing the slides in 3% hydrogen peroxide for 10 minutes. The sections were then incubated with mouse monoclonal primary antibody against nestin (Santa Cruz Biotechnology) in a dilution of 1:100 for 2 hours. The slides were washed and treated with biotin link (Bio SB, Santa Barbara, CA, USA) for 10 minutes. Subsequently horseradish peroxidase enzyme was applied for 10 minutes, and the slides were washed. Freshly prepared diaminobenzidine (DAB) chromogen was used for localizing the antibody binding. Finally, the sections were counter-stained with Mayer’s hematoxylin, mounted, and viewed under a microscope. Mouse kidney tissues, which stain intensely for nestin in the podocytes, were used as positive control.^21^ Negative controls were treated in the same manner but omitting the primary antibody.

### Histological Analysis

Nestin immunostaining was assessed in the entire area available in both tumor and non-tumor epithelium. Cells presenting brown staining in the cytoplasm of epithelial/tumor cells were considered positive for nestin. The immunostained sections were scored according to the percentage of stained cells in three representative areas under ×200 magnification. The staining was assessed according to the method proposed by Ravindran and Devaraj^22^ as follows: 0 (negative), i.e. no staining or staining in less than 5% of cells; 1^+^ (mild), staining in 5%–15% of cells; 2^+^ (moderate), staining in 16%–25% of cells; and 3^+^ (intense), staining in >25% of cells. The staining was also graded as negative (includes categories 0 and 1^+^) and positive (includes categories 2^+^ and 3^+^) for clinico-pathological correlation.

Similarly, in the connective tissue, three separate high-power fields with the highest number of vessels were assessed for counting nestin-positive blood vessels. All stained endothelial cells or cell clusters were counted as one vessel. The mean value for each sample was used for statistical analysis.

### Statistical Analysis

All calculations were performed using the Statistical Package for the Social Sciences (SPSS) version 21 (Chicago, IL, USA). Shapiro–Wilk’s test was used to analyze the distribution pattern. The Kruskal–Wallis test, Student’s *t* test, and the Mann–Whitney *U* test were used to compare the expression of nestin between various groups, whereas the association between nestin expression and clinico-pathological factors was analyzed using the chi-square test. A *P* value of <0.05 was considered to be significant.

## RESULTS

### Immunoblot Analysis

**Figure 1:**
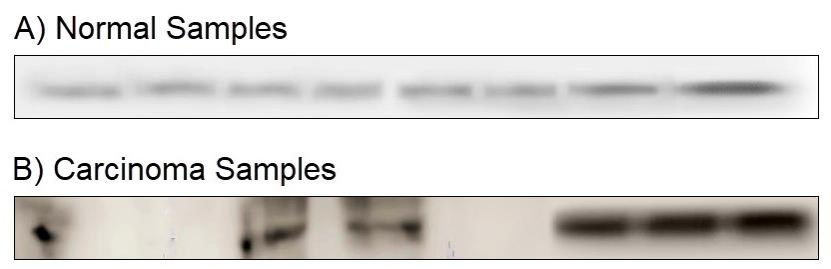
Western Blot Analysis for Nestin Showing Expression for Normal (A) and OSCC (B) Samples. The OSCC samples show an increased expression and intensity compared to normal samples; this reached statistical significance.

The protein band observed in the molecular weight range of 190–200 kDa identified by the primary anti-human nestin antibody was confirmed to be nestin protein. All the normal samples showed faint positivity with thin bands. Six out of 8 OSCC samples showed higher expression of nestin than NOM samples, with a dark thick band; 2 OSCC samples failed to show positive expression for nestin (Figure 1). The mean expression of OSCC samples was found to be six times higher than the NOM samples, and statistical significance was reached. These results showed that nestin is commonly expressed at low levels in normal mucosa but is elevated in the oral cancer tissues.

### Immunohistochemical Analysis

Nestin expression in the keratinocytes is detailed in Table 1.

**Table 1:** Expression of Nestin in the Keratinocytes of Normal Mucosa, Leukoplakia, and Carcinoma Samples.

Type and No. of Samples	Nestin			
	0	1+	2+	3+
Normal (*n*=10)	5	5	0	0
Leukoplakia (*n*=26)	9	11	6	0
Carcinoma (*n*=30)	18	10	2	0

0 (negative), no staining or staining in less than 5% of cells; 1^+^ (mild), staining in 5%–15% of carcinoma cells; 2^+^ (moderate), staining in 16%–25% of cells; and 3^+^ (intense), staining in >25% of cells.

**Figure 2:**
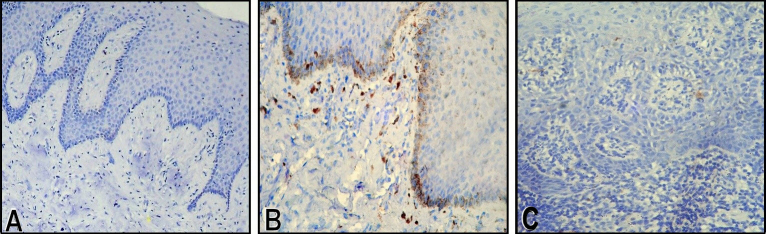
Representative Photomicrographs of Nestin Immunoreactivity. **A:** Normal mucosa; **B:** leukoplakia without dysplasia; **C:** leukoplakia with severe dysplasia (magnification ×100).

**Figure 3:**
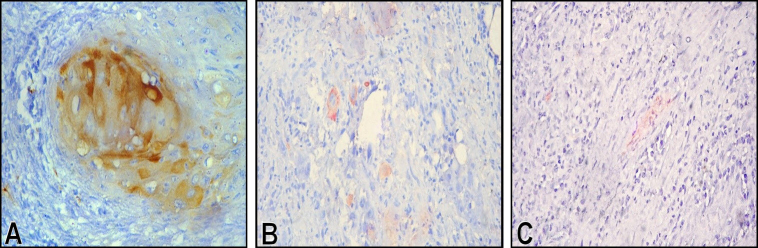
Photomicrographs of OSCC Samples Showing Nestin Expression. **A:** Well differentiated OSCC; **B:** moderately differentiated OSCC; **C:** poorly differentiated OSCC (high-power view).

Nestin expression was confined to the basal layer of the epithelium in NOM samples, whereas it was also seen in the suprabasal layers in leukoplakia samples (Figure 2). In nestin-positive OSCC samples, both peripheral and central cells of the tumor islands showed positivity (Figure 3). However, the difference was not statistically significant.

The leukoplakia group was further sub-divided into leukoplakia without dysplasia, leukoplakia with mild/moderate dysplasia, and leukoplakia with severe dysplasia, and the mean expressions of nestin were 1.3±0.48, 0.9±0.88, and 0.17±0.41, respectively. Statistical analysis among the leukoplakia groups found a highly significant difference at *P*<0.05. Intergroup comparison showed significant variation between leukoplakia without dysplasia and severe dysplasia. No statistical significance was found between other groups of leukoplakia.

Among the 30 cases of OSCC, 2 cases (7%) showed moderate expression, 10 cases (33%) showed mild expression, and the remaining 18 cases (60%) failed to express nestin. The expression of nestin was found to be increased in well differentiated OSCC compared to moderately/poorly differentiated tumors (Figure 3). Furthermore, intergroup comparison showed a statistically significant difference between well differentiated OSCC and poorly differentiated OSCC and also between well differentiated OSCC and moderately differentiated OSCC.

### Nestin Expression and Tumor Invasion Pattern

Oral squamous cell carcinomas were sub-grouped based on the pattern of tumor invasion according to Bryne et al.^23^ Two cases (7%) showed pattern 1 invasion, 4 cases (14%) showed pattern 2 invasion, 14 cases (46%) showed pattern 3 invasion, and 10 cases (33%) showed pattern 4 invasion. Comparison of nestin expression in the keratinocytes among the various invasion patterns showed no statistical significance.

### Nestin Expression and Clinico-pathological Parameters

No significant correlation was found between age, sex, the site, histological grading, or tumor invasion pattern and nestin expression in the samples of leukoplakia (Table 2) and OSCC (Table 3).

**Table 2:** Association of Nestin Expression in the Keratinocytes of Leukoplakia Samples with Clinical and Pathological Factors (Chi-square Test).

Factor	No. of Patients	Nestin *n* (%)		*P* Value

		Negative (*n*=20)	Positive (*n*=6)	
**Age**				
<60 y	20	14 (70)	6 (30)	0.126
≥60 y	6	6 (100)	0 (0)	

**Gender**				
Male	18	12 (66.7)	6 (33.3)	0.063
Female	8	8 (100)	0 (0)	

**Site**				
Tongue	1	1 (100)	0 (0)	0.803
Buccal mucosa	18	14 (77.8)	4 (22.2)	
Floor of the mouth	7	5 (71.4)	2 (28.6)	

**Dysplasia status**				
No dysplasia	10	7 (70)	3 (30)	0.508
Dysplasia	16	13 (81.2)	3 (18.8)	

Negative includes categories 0 and 1^+^, and positive includes categories 2^+^ and 3^+^.

**Table 3:** Association of Nestin Expression in the Keratinocytes of OSCC Samples with Clinical and Pathological Factors (Chi-square Test).

Factor	No. of Patients	Nestin *n* (%)		*P* Value

		Negative (28)	Positive (2)	
**Age**				
<60 y	17	16 (94.1)	1 (5.9)	0.844
≥60 y	13	12 (92.3)	1 (7.7)	

**Gender**				
Male	16	15 (93.8)	1 (6.2)	0.922
Female	14	13 (92.9)	1 (7.1)	

**Site**				
Tongue	1	1 (100)	0 (0)	0.651
Buccal mucosa	17	15 (88.2)	2 (11.8)	
Palate	4	4 (100)	0 (0)	
Floor of the mouth	8	8 (100)	0 (0)	

**Tumor Grade**				
Well differentiated	10	8 (80)	2 (20)	0.117
Moderately differentiated	10	10 (100)	0 (0)	
Poorly differentiated	10	10 (100)	0 (0)	

**Tumor Invasion Pattern**				
Pattern 1	2	2 (100)	0 (0)	0.388
Pattern 2	4	3 (75)	1 (25)	
Pattern 3	14	13 (92.9)	1 (7.1)	
Pattern 4	10	10 (100)	(0.0)	

Negative includes categories 0 and 1^+^, and positive includes categories 2^+^ and 3^+^.

### Nestin Expression in the Connective Tissue

To establish a link between nestin expression and neoangiogenesis, nestin-positive blood vessels were observed in cases of NOM, leukoplakia, and OSCC. There were no immunopositive blood vessels in the NOM samples. However, leukoplakia and OSCC samples showed nestin-positive vessels abundantly near the sub-epithelial area and invasive front of the tumor, respectively. The mean expression of nestin in the blood vessels in NOM, leukoplakia, and OSCC were 0, 1.87±1.58, and 2.65±1.67, respectively, and a statistically significant difference was reached. When an intergroup comparison was made, a significant difference was found between NOM and leukoplakia, and between NOM and OSCC samples. However, no significant difference was observed between leukoplakia and OSCC samples.

## DISCUSSION

Nestin, a type VI IF protein, which was originally described as neural stem cell marker, is highly specific to neural stem cells. Nestin is expressed during early stages of development, and its expression is downregulated upon tissue differentiation and, in course, is replaced by other tissue-specific IF proteins. Interestingly, nestin expression is re-induced during various regenerative and degenerative conditions in the fully differentiated tissues.^19^ Several experimental studies on human solid tumors including OSCC have found higher levels of nestin expression in the tumor cells. Nestin expression is also observed in the endothelial cells of adult tissues that replenish by angiogenesis and in the endothelium of vascular neoplasms and cancers, suggesting that nestin is a marker for angiogenesis.^24^

Till now, only a few reports address the role of nestin in oral carcinogenesis and neoangiogenesis. Hence, to address this issue, the present study observed nestin expression in samples of normal mucosa, leukoplakia, and OSCC using western blot and immunohistochemical analysis. The preliminary study was undertaken to quantify the nestin expression in the normal mucosa and OSCC samples by immunoblotting technique. The present data demonstrated that nestin expression was observed in both normal and OSCC samples. However, the expression in OSCC was found to be six times higher than that of NOM samples. These findings were in accordance to the earlier study on breast cancer in which relatively higher levels of nestin expression were demonstrated.^11^

As nestin is a marker of stem cells, observation of its expression in the normal mucosa (a differentiated tissue) may therefore be attributed to the presence of a stem cell population in the basal layer expressing the protein. In oral cancer, a high level of nestin expression has been shown in oral cancer stem cell-like cells.^25^ It has also been established that there is an increase in nestin expression in the endothelial cells of the newly formed blood vessels, including tumor angiogenesis. Therefore, the higher expression of nestin in OSCC observed in western blot could be due to the above-mentioned factors, and currently, it is unknown which—keratinocytes or endothelial cells—contributed to nestin over-expression.

To further validate the finding, an immunohistochemical analysis was performed to observe the localization and expression pattern of nestin in the study samples. In an attempt to trace the earliest change in nestin upregulation, leukoplakia samples were also included for the study. Most of the studies on the nestin expression in the normal samples showed no expression of nestin.^22^,^26^ In the present study, 5 NOM samples showed mild nestin expression in a few cells of the basal layer, supporting the observation of stem cells dispersed among the basal cells of the normal epithelium, which minimally express nestin.

In a total of 26 leukoplakia cases studied, 65% of cases showed positive expression for nestin in the epithelial cells. The intensity and the number of cells expressing nestin were much higher than NOM. The leukoplakia group was further categorized based on the presence of morphologic changes of the cells (dysplasia) into leukoplakia without any dysplastic features (*n*=10), leukoplakia with mild to moderate dysplastic features (*n*=10), and leukoplakia with severe dysplastic features/carcinoma *in situ* (*n*=6). The purpose of such sub-grouping was that, according to earlier studies on dysplasia, the leukoplakic lesions with epithelial dysplasia had higher risk of turning into carcinoma and the rate of malignant transformation increases with the severity of dysplasia.^27^

Interestingly, the present study showed cytoplasmic and membranous expression of nestin, gradually decreasing from leukoplakia without dysplasia to leukoplakia with mild/moderate dysplasia, and further decreased in leukoplakia with severe dysplasia. This finding was in contrast to an earlier study which observed a gradual increase in the expression of nestin from mild/moderate dysplasia to severe dysplasia^22^; however, that study did not include cases of leukoplakia without dysplasia.

With regard to the present data, nestin expression weakened as the severity of dysplasia increased and there was a statistical difference between the leukoplakia cases with and without dysplasia, indicating that nestin is expressed much earlier, even before microscopic changes are evident in cases of leukoplakia. Hence, it may be speculated that nestin expression could be an early event in the carcinogenesis cascade and that it governs the molecular events that are initiated even before its clinical presentation.

Studies on nestin expression in carcinoma samples are inconclusive and contradictory, with a highly variable expression of nestin-positive tumor cells among various human cancers. Tumor cells expressing nestin were found most frequently in cervical carcinoma (100%),^28^ followed by lung carcinoma (86.5%),^12^ gliomas (82.4%),^15^ prostate cancer (75%),^29^ pancreatic ductal adenocarcinoma (30%),^14^ breast carcinoma (27.33%),^11^ nasopharyngeal carcinoma (2%),^26^ and the lowest being 0% in colorectal carcinoma.^30^

In the present study, only 12 out of 30 (40%) OSCC cases showed nestin positivity in the cytoplasm of the tumor cells. Among the 30 OSCC cases, the expression of nestin was found to be decreased and inversely associated with loss of differentiation. This observation was in contrast to the study by Ravindran and Devaraj,^22^ who observed a gradual increase in nestin expression as the tumor grade became less differentiated. A possible reason for such varied expression of nestin may be due to the difference in tissue-specific progenitor cells and varying phenotype of tumor cells.

The present study could not correlate nestin expression with clinico-pathological factors. Even though nestin-positive tumor cells were observed in various invasion patterns of OSCC, no statistical significance was observed.

Tumor angiogenesis is an important factor in the proliferation and metastasis of neoplasms. The degree of tumor angiogenesis is associated with clinical outcome, as the angiogenic properties correlate with tumor aggressiveness and metastasis.^30^ Evidence also shows that nestin is a vascular marker which transiently appears in undifferentiated endothelial cells, whereas it is not seen in mature vasculature; thus it could represent a marker for newly formed endothelial cells.^31^

Therefore, an attempt was made to determine the status of the newly formed blood vessels by observing nestin expression in the endothelial cells. To the best of our knowledge, this is the first study that has observed the expression of nestin in the blood vessels in the presence of oral carcinogenesis. Out of 10 NOM samples, none of the cases exhibited nestin positivity in the blood vessels, which is in accordance with the fact that nestin expression is seen only in newly formed blood vessels, and such new blood vessel formation occurs only in the walls of the endometrium under physiological conditions. Among the leukoplakia samples, nestin-positive blood vessels were visualized in the sub-epithelial connective tissue. This supports the perspective that angiogenesis starts at a much earlier stage in oral carcinogenesis, especially at the precancerous stage.^31^ In the OSCC samples, nestin-positive blood vessels were prominently observed in the invasive front and in the peri-tumoral regions. This finding is in concordance to a previous study which observed an increase in the expression of VEGF, a potent angiogenesis marker from NOM, through dysplasia, to OSCC, and also suggesting that neoangiogenesis initiates at an early stage during oral carcinogenesis.^32^

Based on these observations, it becomes strongly evident that nestin can be considered as a reliable marker of tumor angiogenesis. In addition, nestin can also be utilized to selectively reflect the vascular density induced by the tumor, which, in turn, may influence the metastasis of tumor cells.

To summarize, there is an upregulation of nestin expression in leukoplakia and OSCC samples, as determined by western blot and immunohistochemical techniques. Although a statistically significant difference was found between most of the study groups, it deserves additional evaluation with a larger biological sample and follow-up data for malignant transformation of leukoplakia cases.

At a minimum, our study data support the notion that nestin plays a brief but pivotal role in oral carcinogenesis and may be a valuable marker for neoangiogenesis in oral cancer. Nestin expression in leukoplakia samples suggests that it could be an early event in the multistage carcinogenesis of oral epithelium.

## Abbreviations

APES: 3-aminopropyltriethoxysilane
CSC: cancer stem cell
DAB: diaminobenzidine
IF: intermediate filament
MVD: microvascular density
NOM: normal oral mucosa
OSCC: oral squamous cell carcinoma
PMD: potentially malignant disorders
PVDF: polyvinylidene difluoride
TBST: tris buffered saline with tween.
